# 

**Published:** 1875-07-01

**Authors:** 


					﻿No. XIII.
JULY 1, 1876.
Vol. XVI.
THE
MIDICAL EXAMINER:
• A
Stmi-ffloirfhlg ilfounral of fflotliral Sciences.
EDITED BY
N. S. DAVIS, M. D., and F. H. DAVIS, M. D.
Subscription Price, Three Dollars per' Annum, in advance.
Single Numbers, Fifteen Cents.
CHICAGO.
PTT BLISIIM) AT 65 E. RANDOLPH STREET.
				

